# The Development of the College Students' Experience of Family Harmony Questionnaire (CSEFHQ)

**DOI:** 10.3389/fpsyg.2021.658430

**Published:** 2021-04-12

**Authors:** Qisheng Zhan, Qin Wang

**Affiliations:** ^1^School of Education, Tianjin University, Tianjin, China; ^2^Institute of Psychology, Tianjin University, Tianjin, China

**Keywords:** college students, experience of family harmony, reliability, validity, questionnaire

## Abstract

The experience of family harmony, as an individual's subjective evaluation of harmonious family relations, has an important influence on the development of their physical and mental health. This study aimed to develop the College Students' Experience of Family Harmony Questionnaire that is fit for college students in China. On the basis of literature analysis and survey with questionnaires, five pairs of opposite assessment indexes were constructed in this paper, namely, Atmosphere of family (getting along vs. conflict), Responsibility to housework (undertaking housework vs. refusing housework), Time-sharing (sharing vs. self-isolatedness), Seeking help (help-seeking vs. avoidance), and Supporting family members (support-providing vs. indifference). Items of this questionnaire were collected from investigation, relevant scales, and discussion with experts. Here, 562 college students were selected for the pre-test and 696 for the formal test. The results showed that, except for the dimension of refusing housework, which has been deleted, other dimensions remain unchanged, and the final nine dimensions accounted for 66.03% of variance variation. Furthermore, the result of confirmatory factor analysis indicates that the model fit well with the data in construct validity [χ^2^/*df* = 2.71, Incremental Fit Index (*IFI*) = 0.90, Tucker–Lewis Index (*TLI*) = 0.89, Comparative Fit Index (*CFI*) = 0.90, root mean square error of approximation (*RMSEA*) = 0.05, standardized root mean square residual (*SRMR*) = 0.05]. The Cronbach's alpha (α) coefficient of this questionnaire was 0.97. The split-half reliability was 0.92, and the test–retest reliability was 0.75 for the total questionnaire. The total score of the questionnaire was significantly positively correlated with the total score of family function, family cohesion, family adaptability, and well-being (*r* = 0.73, 0.71, 0.75, 0.51, respectively, all *p* < 0.01), and it had a significant negative correlation with loneliness (*r* = −0.56, *p* < 0.01). The results showed that the final structure was reasonable, and reliability and validity conformed to the requirements of psychometrics. Therefore, the questionnaire developed in this study can be used as a valid instrument for assessing the experience of family harmony among college students in China.

## Introduction

The value concept of family harmony and prosperity has always been valued by Chinese families. As an ideal state of family relations, family harmony refers to the harmonious coexistence in family life (Yap and Tan, 2011). Family harmony has a very important impact on individual mental health and happy life. Relevant studies show that college students with harmonious families have a higher level of trust than those with quarrelsome families (Zhang, 2016). College students with strong family cohesion and less conflicts show better academic, social, and emotional adaptability during college (Johnson et al., 2010). College students who experienced more family conflict reported more psychological and emotional distress, more depressive symptoms, and poorer social adjustment (Hannum and Dvorak, 2004; Lucas-Thompson and Hostinar, 2013; Rhoades and Wood, 2014). However, the evaluation of family harmony cannot be generalized. Depending on age, experience, personality, and other factors, everyone's views on family harmony may be different. Numerous studies have shown that parents and adolescents may view family relationships in different ways and have a different understanding of family functions (Noller and Callan, 1986; Feldman and Gehring, 1988; Carlson et al., 1991; Grych et al., 1992; Ohannessian et al., 2000). So they do not necessarily have the same idea of family harmony. Feldman and Gehring (1988) pointed out that “family interactions from children's statements and objective assessments are independent. They may be overlapped, but they are both worth studying.” From this point of view, the individual perception and evaluation of family harmony are different from the relatively objective comprehensive evaluation of family harmony. Experience of family harmony is the individual's own subjective evaluation of whether the family relationship is harmonious or not.

Through searching the literature, we can find that there is no measuring tool for family harmony in foreign countries because family harmony is a special topic based on Chinese cultural characteristics, while it is more commonly referred to as healthy family, strong families, happy families, stable families, successful families, optimal families, well-functioning families, and so on in the context of western culture (Wolcott, 1999; Siu and Shek, 2005; Ip, 2014; Fauziah, 2020). Although, in essence, these families also aim to achieve the goal of family harmony and happiness, there are still differences in specific standards and denotation between these families and harmonious families (Yang and Liu, 2008).

The family-related measurement tools developed abroad mainly include Family APGAR Questionnaire (Smilkstein, 1978), Family Environment Scale (Moos and Moos, 1981), Family Adaptability and Cohesion Evaluation Scale (FACES; Olson et al., 1982), Family Assessment Device (FAD; Kabacoff et al., 1990), Self-Report Family Inventory (Beavers et al., 1985). Some domestic scholars have also developed Chinese Family Assessment Instrument (Siu and Shek, 2005; Shek and Ma, 2010; Mellor et al., 2014), Relationship-Specific Chinese Family Assessment Instrument (Liu et al., 2011). Although these measurement tools are related to the content of family harmony, they mainly focus on the family function, and the emphasis is not family harmony.

At present, the main measuring tools for family harmony in China are as follows: (1) Harmonious family evaluation system constructed by evaluating 1,200 families in urban and rural areas of Shanghai (Xu, 2009); (2) Family Harmony Scale and the corresponding five-item simplified version scale, FHS-5 (Kavikondala et al., 2016); (3) Family harmony scale for adolescents (Li, 2016). However, there are also limitations in using the above scales to measure college students' experience of family harmony. First of all, the measurement of family harmony of the first two is a comprehensive assessment of family harmony by different members of the family (including husband, wife, father, mother, children, grandparents, siblings, etc.). Fang et al. (2004) pointed out that most previous studies require a member of the family to evaluate the family function. In fact, this is based on the premise that all family members have the same perception of the family function. Therefore, this kind of evaluation is not necessarily in line with reality, and the evaluation results are general and depersonalized. In addition, although the latter is developed for teenagers in the family, it similarly emphasizes the general form of family interaction. There is still a lack of description of the perspective of individual experience. For example, the items in the questionnaire such as “good health of family members, no major diseases” and “high quality of elders in the family” still focus on the relatively objective description of the family like many other similar family assessment instruments (Shek and Ma, 2010), rather than the subjective harmonious feeling and experience of the individual. Therefore, none of the above scales can accurately evaluate the college students' experience of family harmony. A person's cognitive assessment and perception of the meaning of life events are very important, and relevant studies have shown that external support can have a positive impact on the individual only after it is perceived and recognized by the individual (Rutter, 1981; Li and Yin, 2015). Therefore, the experience of family harmony, as the perception of whether the family is harmonious or not, is more important for the development of his or her physical and mental health. However, previous studies have not dealt with the theme of college students' experience of family harmony. On the one hand, the development of measuring tools for college students' family harmony can enrich relevant research in the field of family harmony and help college students to further understand their cognition of family relations. On the other hand, they can understand their experiences of family relationship. And this study can provide more reference information for family therapy and carry out more targeted work. Therefore, the purpose of the current study was to develop an instrument for measuring the family harmony experience of college students in China on the basis of the existing research and test it from the perspective of psychometrics.

## Method

### Participants

Sample 1: Here, 600 college students from a University in Tianjin were selected as subjects. From them, 562 valid questionnaires were collected, with an effective recovery rate of 94%. Among the valid samples, there were 222 freshmen, 119 sophomores, 116 juniors, and 105 seniors (including 309 males and 253 females). The ages of the students ranged from 17 to 23 (*M* = 19.35, *SD* = 1.39). Sample 1 is used for item analysis and exploratory factor analysis (EFA) of the initial questionnaire.

Sample 2: Here, 800 college students from a University in Tianjin were selected, and 696 valid questionnaires were obtained. The effective recovery rate was 87%. Among the valid samples, there were 271 freshmen, 169 sophomores, 139 juniors, and 117 seniors (including 350 males and 346 females). The ages of the students ranged from 17 to 23 (*M* = 19.30, *SD* = 1.35). Sample 2 was used for confirmatory factor analysis (CFA), construct validity analysis, and internal consistency reliability analysis of the formal questionnaire.

Sample 3: Here, 618 college students from a University in Tianjin were selected, and 519 valid questionnaires were obtained. The effective recovery rate was 83.98%. Among the valid samples, there were 230 freshmen, 127 sophomores, 98 juniors, and 64 seniors (including 280 males and 239 females). The ages of the students ranged from 16 to 23 (*M* = 19.15, *SD* = 1.27). Sample 3 was used to analyze the criterion-related validity of the formal questionnaire.

Sample 4: Here, 248 college students were selected from sample 3 and retested 2 months later. There were 223 valid questionnaires, with an effective recovery rate of 89.92%. Among the valid samples, there were 154 freshmen, 50 sophomores, 11 juniors, and eight seniors (including 100 males and 123 females). This group ranged from 17 to 23 years of age (*M* = 18.96, *SD* = 1.03). Sample 4 was used to analyze the test–retest reliability of the formal questionnaire.

Chinese families are traditionally all heterosexual families, and there are no homosexual families by law. So all of the college students were from heterosexual families. Among them, 618 college students were investigated in this study, among which 35 were from single-parent families, 577 were from non-single-parent families, and six were missing. And there was no significant difference in the total score of harmony between single-parent and non-single-parent families (*p* > 0.05).

### Procedure of Development of the Questionnaire

#### Construction of Dimensions

We have drawn up an open-ended questionnaire on the experience of family harmony, which requires college students to write three items of the experience of family harmony and family disharmony according to their own actual situation. Here, 327 subjects were distributed in a University in Tianjin, including 171 males and 156 females. All the items were collected and sorted out to be 981 items of the experience of family harmony and 976 items of the experience of family disharmony, and the survey results were classified and sorted out. Then, combined with the relevant theories and measurements at home and abroad, it initially formed five aspects: Atmosphere of family, Responsibility to housework, Time-sharing, Seeking help, Supporting family members (ARTSS). Considering the multi-perspective evaluation of college students' experience of family harmony, each aspect consists of the opposite bipolar perspectives to construct specific dimensions and finally form 10 subdimensions. They include getting along vs. conflict, undertaking housework vs. refusing housework, sharing vs. self-isolatedness, help-seeking vs. avoidance, and support-providing vs. indifference, respectively.

#### Preparation of the Items

First of all, the representative items were selected according to the open-ended questionnaire. For example, “There is always full of laughter among my family members at home” belongs to “Getting along.” “My family members often quarrel with each other” belongs to “Conflict.” “We share interesting stories together” belongs to “Sharing.” “We don't have enough time to get along and communicate with each other” belongs to “Self-isolatedness.” “I can help my family members when they are in trouble” belongs to “Support-providing.” “My family and I don't care about each other” belongs to “Indifference.” “I communicate with my family members as soon as possible when something happens to me” belongs to “Help-seeking.” “There's no one to talk about my pain at home” belongs to “Avoidance.” “We don't evade the responsibility of housework” belongs to “Undertaking housework.” A total of 29 items were extracted from the open-ended questionnaire of college students.

Secondly, some items originated from Family Environment Scale, FACES, FAD, The Chinese version of FAD, Family APGAR Questionnaire, and Family Harmony Scale. For example, after referring to the item “I am satisfied with the amount of time my family and I spend together” in the Family APGAR Questionnaire, we modified this item as “I am very satisfied that my family members spend time with me.” Referring to the items in the Family Harmony Scale, “Family members listen to each other's opinions,” we modified this item as “We will listen to each other's opinions when we meet problems” and so on. There are 34 items extracted from the above six questionnaires.

Thirdly, an expert group of four psychologists is formed to discuss whether each item accurately expresses the meaning represented by the corresponding dimensions and whether the expression is appropriate, so as to make appropriate deletions and modifications. For example, the objects of the questionnaire are the college students in a family, not all family members, so the description about the subjects of some items in the existing scale has been modified, such as “family members take the initiative to talk to family members” being modified as “I will take the initiative to talk to family members” and “Other people will pay attention when family members encounter troubles” being modified as “I will pay attention to family members when they encounter troubles.” Finally, 63 items about the experience of family harmony were obtained, and the initial questionnaire was formed. Among them, there are nine items of getting along, seven items of conflict, nine items of undertaking housework, three items of refusing housework, eight items of sharing, five items of self-isolatedness, six items of help-seeking, six items of avoidance, seven items of support-providing, and three items of indifference. The dimensions of the initial questionnaire and the specific sources of all items are shown in Table 1.

**Table 1:** Dimensions and items of the initial questionnaire for college students' experience of family harmony.

**Dimension**	**Item**	**Source of the item**
Getting along	Q1. Every member in my family is free to express his/her opinions.	3
	Q20. My family members can be modest to each other when there is a conflict in the family.	3
	Q21. My family members always get along with each other.	2
	Q38. I feel that everyone in my family is backing each other up.	1
	Q39. My family members don't have to be careful when they communicate with each other.	Modified from 1
	Q53. I don't feel stressed at home.	Modified from 1
	Q54. My family members love each other.	1
	Q61. There is always full of laughter among my family members at home.	1
	Q63. We seldom have family conflicts.	1
Conflict	Q2. I feel like the atmosphere at home is depressing and suffocating.	1
	Q19. My family members have a cold war with each other.	1
	Q22. My family members often quarrel with each other.	1
	Q37.I feel like not to stay at home.	1
	Q40. My family members complain about each other when things go wrong.	1
	Q52. My family members are seldom gentle and considerate to each other.	5
	Q55. My family members often blame and criticize each other.	2
Undertaking housework	Q9. Do it together if something needs to be dealt with at home	3
	Q12. We can share the housework together.	Modified from 4
	Q29. We will discuss the division of housework.	Modified from 4
	Q31. We take turns to share different housework in the family.	3
	Q46.We all share family obligations.	Modified from 3
	Q47. We are willing to spend a lot of energy doing things at home.	Modified from 2
	Q58. We don't evade the responsibility of housework.	1
	Q59. Everyone in my family does his/her job.	1
	Q62. We do housework together.	1
Refusing housework	Q10. We complain to each other that the other side did too little housework.	Modified from 4
	Q11. The housework of our family focuses on individual people.	5
	Q30. Few people volunteer to do something at home.	2
Sharing	Q7. We participate in things we are all interested in.	4
	Q14. We will discuss and consult together when we encounter problems.	Modified from 4
	Q27. We'll show each other our love.	Modified from 4
	Q33. I am very satisfied that my family members spend time with me.	Modified from 6
	Q44. We will listen to each other's opinions when we meet problems.	Modified from 7
	Q48. I will try my best to spend time with my family members.	1
	Q57. My family members take part in recreational activities together.	Adapted from 3
	Q60. We share interesting stories together.	1
Self-isolatedness	Q8. We don't express our love for each other.	5
	Q13. We prefer to do things separately rather than with the whole family.	3
	Q28. I seldom consider other family members' opinions when I do things.	Modified from 2
	Q32.We don't have enough time to get along and communicate with each other.	Modified from 1
	Q45. There is little time for my family members to spend time with each other.	1
Help-seeking	Q3. I can get comfort and help at home when I encounter difficulties.	1
	Q18. I will take the initiative to talk to my family.	Modified from 3
	Q23. I can tell my family about my difficulties and troubles.	Modified from 2
	Q36. I communicate with my family members as soon as possible when something happens to me.	1
	Q51. I will discuss the solution with my family if I have a problem.	Modified from 4
Avoidance	Q4. There's no one to talk to about my pain at home.	1
	Q17. It's hard to talk to my family when I come across something that makes me sad.	Modified from 5
	Q24.I don't talk to my family when I'm angry.	5
	Q35. I choose to take it alone when I have something to worry about.	1
	Q42. I never tell my family what's on my mind.	4
	Q50.I don't tell my family what happened.	2
Support-providing	Q5.I will pay attention to my family members when they are in trouble.	Modified from 4
	Q16. We can support each other in times of crisis.	Modified from 4
	Q25. My family can accept and support it when I engage in new activities.	Modified from 6
	Q34. I will support the ideas or decisions of other family members.	1
	Q41. My family would like to listen to my opinions and ideas patiently and support me as much as possible.	1
	Q43. I can help my family members when they are in trouble.	Modified from 1
	Q49. I will care for my family members.	Modified from 1
	Q56. I can give warmth and comfort to my family members when they need it.	Modified from 1
Indifference	Q6. I'm self-centered and I don't care about my family.	Modified from 4
	Q15. My family and I don't care about each other.	Modified from 1
	Q26. My family members only care about themselves and ignore the family.	Modified from 1

*1, Open-ended survey of college students; 2, Family Environment Scale; 3, Family Adaptability and Cohesion Evaluation Scale; 4, Family Assessment Device; 5, the Chinese version of FAD; 6, Family APGAR Questionnaire; 7, Family Harmony Scale*.

#### Formation of the Questionnaire

The subjects of sample 1 were measured with the initial questionnaire of college students' experience of family harmony. In the item analysis, one item with poor differentiation is deleted according to the standard that the correlation coefficient between each item and the total score is <0.4 (Wu, 2010). The measurement results of the remaining 62 items were analyzed with EFA. Such items would be deleted if factor loadings were <0.4, the number of the items in a factor was <3, an item has excessive loading on multiple factors, and having a discrepancy of meaning between the item and the related factor (Wu, 2010). Finally, a formal questionnaire was formed, including 56 items in nine dimensions. The subjects of sample 2 were measured with the formal questionnaire. CFA, construct validity analysis, and internal consistency reliability analysis were carried out, and criterion-related validity was analyzed after measurement of sample 3. The participants in sample 4 were retested 2 months later for test–retest reliability analysis (see the Results section for details).

### Instruments

#### Open-Ended Questionnaire for College Students

We have prepared an open-ended questionnaire for college students' experience of family harmony, which contains two questions: (1) How do you experience family harmony? Please write at least three items. (2) How do you experience family disharmony? Please write at least three items.

#### Initial Questionnaire on College Students' Experience of Family Harmony

The self-designed initial questionnaire contains five groups of evaluation indicators with bipolarity, namely, a total of 10 dimensions, which are getting along, conflict, undertaking housework, refusing housework, sharing, self-isolatedness, help-seeking, avoidance, support-providing, and indifference. The questionnaire consists of 63 items, including 24 reverse scoring items. Scoring of each item in the College Students' Experience of Family Harmony Questionnaire (CSEFHQ) is based on a 4-point Likert scale, which ranges from 1 to 4 (1 = strongly disagree, 2 = disagree, 3 = agree, 4 = strongly agree), and a reverse score was given to 24 items reflecting experience of family disharmony.

#### Formal Questionnaire on College Students' Experience of Family Harmony

The self-designed formal questionnaire consists of nine dimensions, including getting along, conflict, undertaking housework, sharing, self-isolatedness, help-seeking, avoidance, support-providing, and indifference. This questionnaire has 56 items, each with the same scoring method as the initial questionnaire, including 21 reverse scoring items. The higher the final score is, the higher the experience of family harmony is.

#### Criterion Questionnaire

(1) **Family APGAR Questionnaire**

The Family APGAR Questionnaire was compiled by Smilkstein (1978) to evaluate Adaption, Partnership, Growth, Affection, and Resolve of family function. The questionnaire has five items. Respondents use a 3-point Likert-type scale (ranging from *never* to *often*) for each item. The higher the score is, the higher the family support is. In this study, the Cronbach's alpha reliability of the questionnaire is 0.86.

(2) **Family Adaptability and Cohesion Evaluation Scale**

The FACES II was compiled by Olson et al. (1982). Fei et al. (1991) translated and revised the FACES II-CV. There were 30 items in the evaluation of family intimacy and family adaptability. Respondents use a 5-point Likert-type scale (ranging from *never* to *always*) with each item. In this study, the Cronbach's alpha reliability of family intimacy, family adaptability, and the total scale was 0.88, 0.88, 0.94, respectively.

(3) **Well-Being Scale**

The Index of Well-Being (IWB) Scale, compiled by Campbell (1976), is used to measure the degree of happiness experienced by individuals at present, including the overall emotion index and life satisfaction index, a total of nine items. Scores on this scale are based on nine items with a 7-point Likert scale. The higher the score is, and the higher the happiness is. In this study, the Cronbach's alpha reliability of the scale is 0.91.

(4) **Loneliness Scale**

The UCLA Loneliness Scale, compiled by Russel et al. (1978), is used to evaluate individual loneliness caused by the gap between the desire for social communication and the actual level. Scores on this scale are based on 20 items with a 4-point Likert scale ranging from *never* to *often*. The higher the total score is, the higher the loneliness is. In this study, the Cronbach's alpha reliability of the scale is 0.91.

### Data Processing

After the questionnaire was collected uniformly, the data were input into Statistical Package for the Social Sciences (SPSS) software. The researchers used SPSS 21.0 and AMOS 22.0 to analyze the data.

## Results

### Item Analysis of the Initial Questionnaire

Two methods are used in the item analysis. The first is the critical ratio method, in which the total scores of the items are sorted from high to low, and the samples are divided into the top 27% and the last 27%, to test whether there is a significant difference between the score of top-score group and that of low-score group on each item, that is, the significance of the item critical value (CR). The results show that the *p*-value of each item is <0.001, and the discrimination degree is ideal. The second is the correlation method. The Pearson correlation coefficient (r) between the scores of each item and the total score of family harmony shows that the 10th item should be deleted because the correlation coefficient is <0.4 (Wu, 2010). However, the correlation coefficients of the remaining 62 items are between 0.40 and 0.75, and all reach a significant level of 0.01. There is a good item differentiation.

### Exploratory Factor Analysis of the Initial Questionnaire

The remaining 62 items in the initial questionnaire were analyzed with EFA. The results showed that the Kaiser–Meyer–Olkin value is 0.963, exceeding the recommended value of 0.6, as suggested by Kaiser (1974). Bartlett's Test of Sphericity (Bartlett, 1950) is also significant (χ2 = 23939.096, df = 1891, *p* < 0.001), which indicated that the set of correlations in the correlation matrix were significantly different from zero and suitable for factor analysis. The principal component analysis with variance maximum rotation method is used to extract common factors, and the number of factors is determined according to the Eigenvalue exceeding 1 (Kim and Mueller, 1978). Deleted items are with reference to the following criteria: (1) factor loading is <0.4; (2) commonality is <0.2; (3) the factor contains <3 items; (4) the loading on multiple factors is too high; (5) the meaning of the item is not consistent with the meaning of the factor (Wu, 2010). After deleting six items (items 9, 11, 25, 30, 41, and 58), the CSEFHQ resulted in the extraction of nine factors consisting of 56 items, and the cumulative variance explanation rate is 66.03%. The factor loading of each item is shown in Table 2.

**Table 2:** Nine factors of the CSEFHQ and their factor loadings (*N* = 562).

**Factors**	**Items**	**Loadings**
F1: Getting along	Q53. I don't feel stressed at home.	0.69
	Q39. My family members don't have to be careful when they communicate with each other.	0.68
	Q54. My family members love each other.	0.68
	Q63. We seldom have family conflicts.	0.67
	Q21. My family members always get along with each other.	0.65
	Q20. My family members can be modest to each other when there is a conflict in the family.	0.63
	Q38. I feel that everyone in my family is backing each other up.	0.63
	Q61. There is always full of laughter among my family members at home.	0.62
	Q1. Every member in my family is free to express his/her opinions.	0.61
F2: Conflict	Q19. My family members have a cold war with each other^▴^.	0.75
	Q40. My family members complain about each other when things go wrong^▴^.	0.73
	Q55. My family members often blame and criticize each other^▴^.	0.72
	Q22. My family members often quarrel with each other^▴^.	0.71
	Q52. My family members are seldom gentle and considerate to each other^▴^.	0.70
	Q37.I feel like not to stay at home^▴^.	0.69
	Q2. I feel like the atmosphere at home is depressing and suffocating^▴^.	0.63
F3: Undertaking housework	Q31. We take turns to share different housework in the family.	0.79
	Q29. We will discuss the division of housework.	0.78
	Q12. We can share the housework together.	0.75
	Q62. We do housework together.	0.74
	Q46. We all share family obligations.	0.67
	Q59. Everyone in my family does his/her job.	0.54
	Q47. We are willing to spend a lot of energy doing things at home.	0.53
F4: Avoidance	Q35. I choose to take it alone when I have something to worry about^▴^.	0.76
	Q42. I never tell my family what's on my mind^▴^.	0.75
	Q24. I don't talk to my family when I'm angry^▴^.	0.71
	Q17. It's hard to talk to my family when I come across something that makes me sad^▴^.	0.69
	Q50. I don't tell my family what happened^▴^.	0.67
	Q4. There's no one to talk about my pain at home^▴^.	0.66
F5: Support-providing	Q49. I will care for my family members.	0.72
	Q5. I will pay attention to my family members when they are in trouble.	0.70
	Q43. I can help my family members when they are in trouble.	0.70
	Q56. I can give warmth and comfort to my family members when they need it.	0.64
	Q16. We can support each other in times of crisis.	0.63
	Q34. I will support the ideas or decisions of other family members.	0.60
F6: Sharing	Q44. We will listen to each other's opinions when we meet problems.	0.58
	Q33. I am very satisfied that my family members spend time with me.	0.58
	Q48. I will try my best to spend time with my family members.	0.58
	Q60. We share interesting stories together.	0.57
	Q57. My family members take part in recreational activities together.	0.55
	Q27. We'll show each other our love.	0.54
	Q14. We will discuss and consult together when we encounter problems.	0.52
	Q7. We participate in things we are all interested in.	0.45
F7: Help-seeking	Q23. I can tell my family about my difficulties and troubles.	0.72
	Q18. I will take the initiative to talk to my family.	0.71
	Q36. I communicate with my family members as soon as possible when something happens to me.	0.67
	Q3. I can get comfort and help at home when I encounter difficulties.	0.65
	Q51. I will discuss the solution with my family if I have a problem.	0.64
F8: Self-isolatedness	Q32. We don't have enough time to get along and communicate with each other^▴^.	0.75
	Q45. There is little time for my family members to spend time with each other^▴^.	0.65
	Q28. I seldom consider other family members' opinions when I do things^▴^.	0.60
	Q13. We prefer to do things separately rather than with the whole family^▴^.	0.59
	Q8. We don't express our love for each other^▴^.	0.59
F9: Indifference	Q15. My family and I don't care about each other^▴^.	0.80
	Q6. I'm self-centered and I don't care about my family^▴^.	0.67
	Q26. My family members only care about themselves and ignore the family^▴^.	0.59

CSEFHQ, College Students' Experience of Family Harmony Questionnaire. F1–F9 represent the nine extracted factors.

▴ *Indicates negative scoring items. Item number originated from initial questionnaire*.

As shown in Table 2, F1 consists of nine items, which belong to the getting along in the theoretical conception; F2 consists of seven items, which belong to the conflict in the theoretical conception; F3 consists of seven items, which belong to the undertaking housework in the theoretical conception; F4 consists of six items, which belong to the avoidance in the theoretical conception; F5 consists of six items, which belong to the support-providing in the theoretical conception. F6 consists of eight items, which belong to the sharing in the theoretical conception; F7 consists of five items, which belong to the help-seeking in the theoretical conception; F8 consists of five items, which belong to the self-isolatedness in the theoretical conception; and F9 consists of three items, which belong to the indifference in the theoretical conception. There is a total of 56 items, including 21 reverse scoring questions.

### Confirmatory Factor of Analysis of the Formal Questionnaire

**Figure 1:**
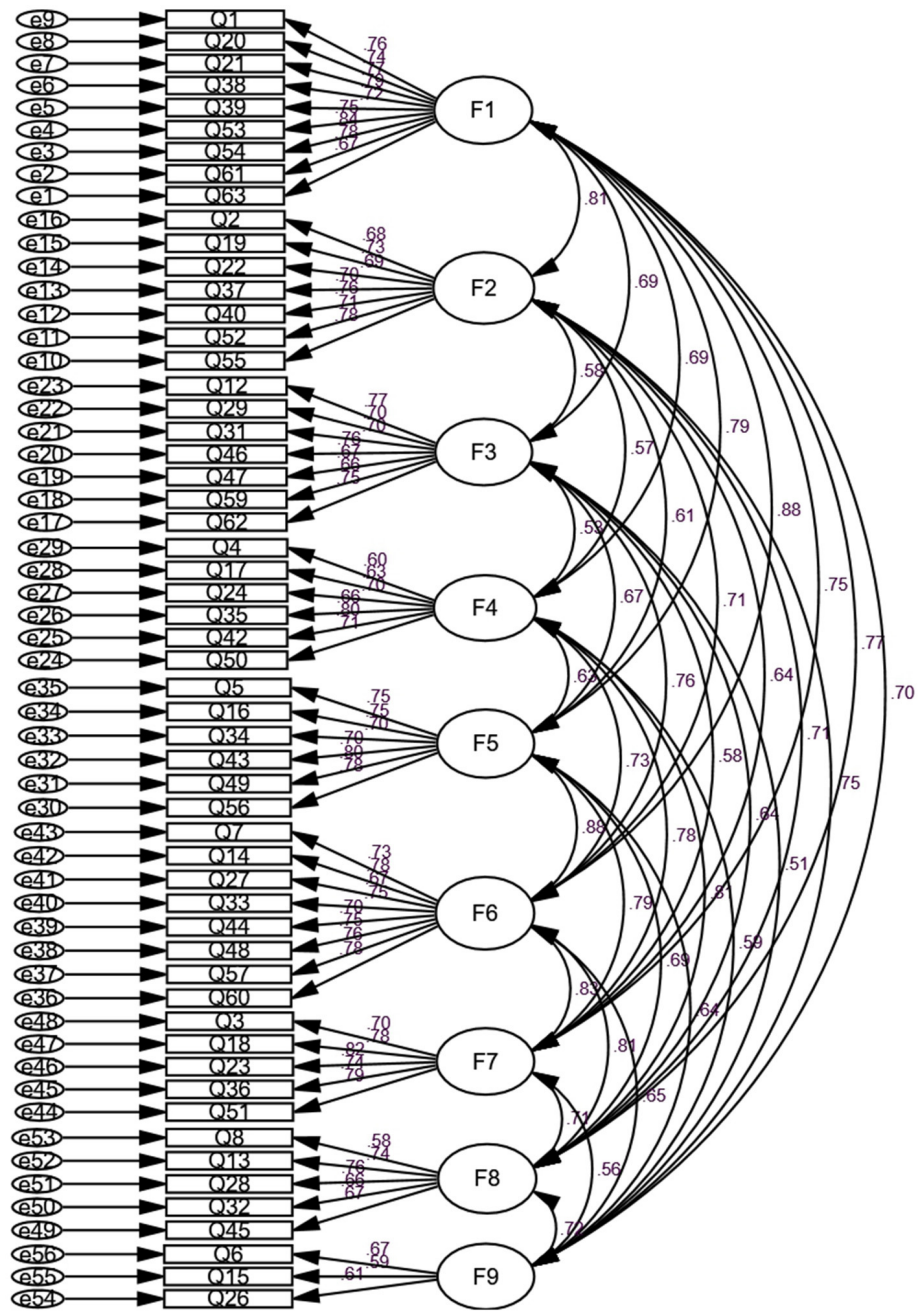
Path diagram and estimated parameter loadings for the nine-factor model of the college students' experience of family harmony questionnaire (CSEFHQ) (*N* = 696). Latent variables (factors F1–F9) are indicated with ovals, and observed variables (items Q1–Q63) are indicated with rectangles.

According to the results of EFA, we decided to run confirmatory factor analysis of the nine factors that consist of 56 items. The CFA was performed using the AMOS 22.0 program. The following commonly used criteria were utilized in evaluating the adequacy of the model: the value of χ^2^/*df* <3 is good. Comparative Fit Index (CFI), Tucker–Lewis Index (TLI), and Incremental Fit Index (IFI) have values ranging from 0 to 1, and the values closer to 1 are much better. The values of root mean square error of approximation (RMSEA) and standardized root mean square residual (SRMR) that are <0.08 indicate that the model fits well on the whole (Wen et al., 2004). The results of this study showed that χ^2^/*df* = 2.71 <3, *p* < 0.001. And the values of *IFI, TLI*, and *CFI* were all >0.85. *RMSEA* = 0.05 < 0.08, *SRMR* = 0.05 < 0.08. Overall, the main fit indexes for the nine-factor model were acceptable (Table 3), indicating that the formal questionnaire structure meets the expectation. In the path diagram of CFA of this model, the standardized factor loading of each item on the factors is > 0.5 and significant (Figure 1). These results show that the formal questionnaire has good construct validity.

**Table 3:** Fit indexes of CFA of the CSEFHQ (*N* = 696).

***χ2***	***df***	***χ2/df***	**IFI**	**TLI**	**CFI**	**RMSEA**	**SRMR**
3,918.99	1,448	2.71	0.90	0.89	0.90	0.05	0.05

*CFA, confirmatory factor analysis; CFI, Comparative Fit Index; CSEFHQ, College Students' Experience of Family Harmony Questionnaire; IFI, Incremental Fit Index; RMSEA, root mean square error of approximation; SRMR, standardized root mean square residual; TLI, Tucker–Lewis Index*.

### Correlation Analysis Among the Scores of Each Factor of the Formal Questionnaire

In order to further test the structural validity of the formal questionnaire, the correlations among the factors of the CSEFHQ and those between the factors and the total score were analyzed. The correlation coefficient between each factor is at a medium or low level, indicating that each factor has the same direction, but there are certain differences. The correlation coefficient between each factor and the total score is at a medium or high level, indicating that each factor has a good consistency with the total questionnaire. The results showed that the correlation coefficients among the factors were between 0.37 and 0.79, and the correlation was significant (*p* < 0.01). The correlation coefficient between each factor and the total score was between 0.61 and 0.91, and the correlation was significant (*p* < 0.01). The correlation between each factor and the total score was higher than that between each factor (Table 4). In summary, the questionnaire has good structural validity.

**Table 4:** The correlation between the scores of factors and the total score of the CSEFHQ (*N* = 696).

	**F1**	**F2**	**F3**	**F4**	**F5**	**F6**	**F7**	**F8**	**F9**
F2	0.72^**^								
F3	0.62^**^	0.50^**^							
F4	0.61^**^	0.50^**^	0.46^**^						
F5	0.69^**^	0.53^**^	0.59^**^	0.55^**^					
F6	0.79^**^	0.63^**^	0.67^**^	0.64^**^	0.78^**^				
F7	0.67^**^	0.56^**^	0.51^**^	0.67^**^	0.69^**^	0.74^**^			
F8	0.64^**^	0.58^**^	0.54^**^	0.66^**^	0.57^**^	0.69^**^	0.60^**^		
F9	0.54^**^	0.57^**^	0.37^**^	0.44^**^	0.48^**^	0.49^**^	0.42^**^	0.50^**^	
CSEFHQ	0.89^**^	0.77^**^	0.75^**^	0.77^**^	0.81^**^	0.91^**^	0.82^**^	0.80^**^	0.61^**^

** *p < 0.01. CSEFHQ, College Students' Experience of Family Harmony Questionnaire*.

### Criterion Validity Analysis of the Formal Questionnaire

The index of family care reflects the subjective satisfaction with the family, and family intimacy is the evaluation of the emotional connection and intimate relationship of family members. In addition, studies have shown that family relationships are closely related to individual happiness and loneliness (Duan, 1996; Deng and Zheng, 2013). Therefore, the Family APGAR Questionnaire, FACES, the IWB scale, and UCLA Loneliness Scale were selected as criterion tools. The results showed that the scores of each factor and the total scores of the CSEFHQ are correlated significantly with measures of APGAR (*r* = 0.73, *p* < 0.01), cohesion (*r* = 0.71, *p* < 0.01), adaptability (*r* = 0.75, *p* < 0.01), well-being (*r* = 0.51, *p* < 0.01), and loneliness (*r* = −0.56, *p* < 0.01). It shows that the criterion validity of the CSEFHQ is good (Table 5).

**Table 5:** Correlation coefficients between factors and criteria of the CSEFHQ (*N* = 519).

	**F1**	**F2**	**F3**	**F4**	**F5**	**F6**	**F7**	**F8**	**F9**	**CSEFHQ**
APGAR	0.66^**^	0.61^**^	0.48^**^	0.52^**^	0.58^**^	0.64^**^	0.68^**^	0.53^**^	0.40^**^	0.73^**^
Cohesion	0.64^**^	0.49^**^	0.59^**^	0.56^**^	0.48^**^	0.70^**^	0.61^**^	0.52^**^	0.35^**^	0.71^**^
Adaptability	0.70^**^	0.56^**^	0.64^**^	0.56^**^	0.52^**^	0.70^**^	0.63^**^	0.53^**^	0.36^**^	0.75^**^
Well-being	0.44^**^	0.39^**^	0.36^**^	0.37^**^	0.44^**^	0.45^**^	0.44^**^	0.40^**^	0.22^**^	0.51^**^
Loneliness	−0.44^**^	−0.45^**^	−0.44^**^	−0.40^**^	−0.52^**^	−0.49^**^	−0.48^**^	−0.45^**^	−0.30^**^	−0.56^**^

** *p < 0.01. CSEFHQ, College Students' Experience of Family Harmony Questionnaire*.

### Reliability Analysis of the Formal Questionnaire

This study examined the internal consistency reliability, split-half reliability, and test–retest reliability of the formal questionnaire. The results show that the Cronbach's alpha coefficient of the total questionnaire is 0.97, and the Cronbach's alpha coefficient of each factor is 0.66–0.91. All these coefficients are >0.60, indicating that the questionnaire has good internal consistency and the reliability was at a good level (Wu, 2009). The split-half reliability of the total questionnaire is 0.92, and the split-half reliability of each factor is 0.67–0.90, indicating that the questionnaire has good equivalence. The test–retest reliability of the total questionnaire is 0.75, and the test–retest reliability of each factor is 0.49–0.72, and correlations are significant. According to relevant criteria, the test–retest reliability is acceptable (Robinson et al., 2010), indicating that the questionnaire has good cross-time stability. Overall, the CSEFHQ has good reliability, and the specific reliability indicators of the questionnaire are shown in Table 6.

**Table 6:** Reliability of the CSEFHQ (Internal consistency and split-half: *N* = 696; Test–retest: *N* = 223).

	**F1**	**F2**	**F3**	**F4**	**F5**	**F6**	**F7**	**F8**	**F9**	**CSEFHQ**
Internal consistency	0.91	0.88	0.88	0.84	0.88	0.90	0.87	0.81	0.66	0.97
Split-half	0.90	0.84	0.81	0.82	0.87	0.89	0.86	0.84	0.67	0.92
Test–retest	0.68^**^	0.63^**^	0.63^**^	0.72^**^	0.60^**^	0.68^**^	0.66^**^	0.62^**^	0.49^**^	0.75^**^

** *p < 0.01. CSEHFQ, College Students' Experience of Family Harmony Questionnaire*.

## Discussion

On the basis of literature analysis and survey with questionnaires and reference to the existing research at home and abroad, this study first constructed five pairs of evaluation indicators of the college students' experience of family harmony with bipolarity. The reason for evaluating college students' experience of family harmony from the perspective of positive and negative experience is that two opposite perspectives can lead to a more real family harmony experience. Thus, it makes the evaluation results more accurate and reliable. At the same time, it can more comprehensively and accurately reflect all aspects of college students' experience of family harmony. Secondly, the initial questionnaire is formed by establishing the questionnaire items through the open-ended questionnaire, reference to the relevant scale, and expert group evaluation. Then, the development of the questionnaire has been finally completed through the pretest, formal test, and retest to test the reliability and validity of the questionnaire.

The developmental process of the whole questionnaire is as follows:

The initial questionnaire consisted of 63 items with 10 dimensions (or factors): getting along, conflict, undertaking housework, refusing housework, sharing, self-isolatedness, help-seeking, avoidance, support-providing, and indifference. According to the results of item analysis, the 10th item Q10 (we complain to each other that the other side did too little correlation) should be deleted because the correlation coefficient is <0.4. Then, EFA was performed, and six items have been deleted. The 11th item Q11 (the housework of our family focuses on individual people) and the 41st item Q41 (my family would like to listen to my opinions and ideas patiently and support me as much as possible) were deleted due to multiple loads. The ninth item Q9 (do it together if something needs to be dealt with at home), 25th item Q25 (my family can accept and support it when I engage in new activities), and 58th item Q58 (we don't evade the responsibility of housework) have been deleted according to matching of the item meaning. The 30th item Q30 (few people volunteer to do something at home) has been deleted because it is inconsistent with the meaning of the factor “self-isolatedness.” The questionnaire remains to have 56 items after seven items were deleted. Since all three items in the factor “refusing housework” had been deleted (Q10, Q11, and Q30), the final formal questionnaire only included nine factors: getting along, conflict, undertaking housework, sharing, self-isolatedness, help-seeking, avoidance, support-providing, and indifference.

Among the nine extracted factors, the two factors of “getting along” and “conflict” explain the highest ratio of variance in EFA. In addition, by conducting a survey with the open-ended questionnaires, it is also found that the highest proportions of family harmony and family disharmony experienced by college students are “family members getting along well” and “family members often have conflicts and disputes,” respectively. This shows that the harmonious coexistence of family members is the most important part of college students' experience of family harmony. Shek and Man-fei (2000) believe that the ideal family in Chinese Confucianism puts great emphasis on family harmony and avoids quarrels. A survey conducted by Shek (2001) in Hong Kong also shows that people place special emphasis on avoiding conflicts in family harmony. Referring to the existing measurement tools related to family harmony (Xu, 2009; Kavikondala et al., 2016; Li, 2016), the dimension of housework responsibility is not involved, so this is a significant feature of the CSEFHQ. Housework is an important part of family life. Family harmony not only is the close and harmonious relationship between family members but also includes the responsibility for family affairs. Family members' division of labor and shared family obligations are one of the elements of family happiness and harmony (Wolcott, 1999; Fauziah, 2020). If housework is concentrated on individual members, it is easy to cause complaints, dissatisfaction, and even disputes. The study of Liu et al. (2015) found that housework is a kind of emotional work and is not simple labor. Sharing housework contributes to the intimacy and harmony of family members, especially husband and wife. Therefore, housework responsibility is of great significance to family harmony, and it is also an important part of college students' experience of family harmony. Help-seeking and avoidance and support and indifference reflect whether college students can get help in their families and whether they can provide support to their families. These are the manifestations of interpersonal interaction among family members, which are similar to the mutuality dimension in the evaluation of Chinese family function by Siu and Shek (2005), emphasizing mutual support of family members. Sharing and self-isolatedness reflect whether college students spend time with their families and share happiness or less than companionship and lack of sharing. Previous studies have shown that family interaction following the sharing model contributes to family harmony (Chuang, 2005), and sharing family time is the core of family harmony and happiness (Lam et al., 2012). Therefore, sharing with family members is also an important part of college students' experience of family harmony. The experience of family harmony has an important influence on the physical and mental development of college students (Johnson et al., 2010; Lucas-Thompson and Hostinar, 2013; Rhoades and Wood, 2014; Cheung et al., 2019), but there is no measurement tool for this aspect at present. So, on the one hand, the development of the CSEFHQ can enrich relevant research in the field of family harmony. On the other hand, it can provide practical guidance for improving college students' experience of family harmony and better provide practical guidance for the development of college students' physical and mental health. However, due to the convenience of sampling, this study only selected the sample of college students in Tianjin but did not select more samples of national college students, so it needs to be further verified and improved in the future. Actually, that belongs to one limitation of this study. If this questionnaire is used in the general population, further research should be conducted. This study focuses on college students' experience of family harmony, and future research can further examine different family roles, such as college students' parents' experience of family harmony. By comparing the evaluation results, we can determine the possible differences of the experience of family harmony based on different perspectives and the influence of these differences on them. Finally, the questionnaire developed in this study is aimed at general and healthy families, and further research is needed if family members have mental illness.

## Conclusion

This study developed an effective instrument to measure college students' experience of family harmony in China. The CSEFHQ with 56 items consists of nine factors: getting along and conflict, help-seeking and avoidance, support-providing and indifference, sharing and self-isolatedness, and undertaking housework. The structure of the questionnaire is reasonable, and the discrimination, reliability, and validity of the items meet the requirements of psychometrics. This study mainly discusses the experience of family harmony among Chinese college students, and the experience of family harmony among other groups besides college students can be further explored. In addition, due to the differences between different cultures, the experience of family harmony and its differences among different groups in the cross-cultural background can be further studied in the future.

## Data Availability Statement

The original contributions presented in the study are included in the article/Supplementary Material, further inquiries can be directed to the corresponding author.

## Ethics Statement

The studies involving human participants were reviewed and approved by The Ethics Committee, Tianjin University. Written informed consent to participate in this study was provided by the participants' legal guardian/next of kin.

## Author Contributions

QZ: construct the framework of the development of this questionnaire (CSEFHQ) and the related idea. QW: carried out this program into effect. All authors contributed to the article and approved the submitted version.

## Conflict of Interest

The authors declare that the research was conducted in the absence of any commercial or financial relationships that could be construed as a potential conflict of interest.
