# Supplementary material for: The Development of the College Students' Experience of Family Harmony Questionnaire (CSEFHQ)
Source: Front Psychol. 2021 Apr 12;12:658430. doi: 10.3389/fpsyg.2021.658430 (PMC8071946; doi:10.3389/fpsyg.2021.658430)
Supplement: Supplementary file 1 [file Data_Sheet_1.PDF]

Appendices

1 The Initial Questionnaire of the College Students' Experience of Family Harmony.

Instructions: Read each of the following statements carefully and tick the number that best represents your true feelings, based on your true experience. 1= Strongly Disagree, 2 = Disagree, 3 = Agree, 4 = Strongly Agree.

Item	Strongly Disagree	Disagree	Agree	Strongly Agree
Q1. Every member in my family is free to express his/her opinions.	1	2	3	4
Q2. I feel like the atmosphere at home is depressing and suffocating.	1	2	3	4
Q3. I can get comfort and help at home when I encounter difficulties.	1	2	3	4
Q4. There's no one to talk about my pain at home.	1	2	3	4
Q5.I will pay attention to my family members when they are in trouble.	1	2	3	4
Q6. I'm self-centered and I don't care about my family.	1	2	3	4
Q7. We participate in things we are all interested in.	1	2	3	4
Q8. We don't express our love for each other.	1	2	3	4
Q9. Do it together if something needs to be dealt with at home	1	2	3	4
Q10. We complain to each other that the other side did too little housework.	1	2	3	4
Q11. The housework of our family focuses on individual people.	1	2	3	4
Q12. We can share the housework together.	1	2	3	4
Q13. We prefer to do things separately rather than with the whole family.	1	2	3	4
Q14. We will discuss and consult together when we encounter problems.	1	2	3	4
Q15. My family and I don't care about each other.	1	2	3	4
Q16. We can support each other in times of crisis.	1	2	3	4
Q17. It's hard to talk to my family when I come across something that makes me sad.	1	2	3	4
Q18. I will take the initiative to talk to my family.	1	2	3	4
Q19. My family members have a cold war with each other .	1	2	3	4
Q20. My family members can be modest to each other when there is a conflict in the family.	1	2	3	4
Q21. My family members always get along with each other.	1	2	3	4
Q22. My family members often quarrel with each other.	1	2	3	4
Q23. I can tell my family about my difficulties and troubles.	1	2	3	4
Q24.I don't talk to my family when I'm angry.	1	2	3	4
Q25. My family can accept and support it when I engage in new activities.	1	2	3	4
Q26. My family members only care about themselves and ignore the family.	1	2	3	4
Q27. We'll show each other our love.	1	2	3	4
Q28. I seldom consider other family members' opinions when I do things.	1	2	3	4
Q29. We will discuss the division of housework.	1	2	3	4
Q30. Few people volunteer to do something at home.	1	2	3	4
Q31. We take turns to share different housework in the family.	1	2	3	4
Q32.We don't have enough time to get along and communicate with each other.	1	2	3	4
Q33. I am very satisfied that my family members spend time with me.	1	2	3	4
Q34. I will support the ideas or decisions of other family members.	1	2	3	4
Q35. I choose to take it alone when I have something to worry about.	1	2	3	4
Q36. I communicate with my family members as soon as possible when something happens to	1	2	3	4

me.				
Q37.I feel like not to stay at home.	1	2	3	4
Q38. I feel that everyone in my family is backing each other.	1	2	3	4
Q39. My family members don't have to be careful when they communicate with each other.	1	2	3	4
Q40. My family members complain about each other when things go wrong.	1	2	3	4
Q41. My family would like to listen to my opinions and ideas patiently and support me as much as possible.	1	2	3	4
Q42. I never tell my family what's on my mind.	1	2	3	4
Q43. I can help my family members when they are in trouble.	1	2	3	4
Q44. We will listen to each others' opinions when we meet problems.	1	2	3	4
Q45. There is little time for my family members to spend time with each other.	1	2	3	4
Q46.We all share family obligations.	1	2	3	4
Q47. We are willing to spend a lot of energy doing things at home.	1	2	3	4
Q48. I will try my best to spend time with my family members.	1	2	3	4
Q49. I will care for my family members.	1	2	3	4
Q50.I don't tell my family what happened.	1	2	3	4
Q51. I will discuss the solution with my family if I have a problem.	1	2	3	4
Q52. My family members are seldom gentle and considerate to each other.	1	2	3	4
Q53. I don't feel stressed at home.	1	2	3	4
Q54. My family members love each other.	1	2	3	4
Q55. My family members often blame and criticize each other.	1	2	3	4
Q56. I can give warmth and comfort to my family members when they need it.	1	2	3	4
Q57. My family members take part in recreational activities together.	1	2	3	4
Q58. We don't evade the responsibility of housework.	1	2	3	4
Q59. Everyone in my family does his/her job.	1	2	3	4
Q60. We share interesting stories together.	1	2	3	4
Q61. There is always full of laughter among my family members at home.	1	2	3	4
Q62. We do housework together.	1	2	3	4
Q63. We seldom have family conflicts.	1	2	3	4

2 The Formal Questionnaire of the College Students' Experience of Family Harmony.

Instructions: Read each of the following statements carefully and tick the number that best represents your true feelings, based on your true experience. 1= Strongly Disagree, 2 = Disagree, 3 = Agree, 4 = Strongly Agree.

Item	Strongly Disagree	Disagree	Agree	Strongly Agree
1. Every member in my family is free to express his/her opinions.	1	2	3	4
2. I feel like the atmosphere at home is depressing and suffocating.	1	2	3	4
3. I can get comfort and help at home when I encounter difficulties.	1	2	3	4
4. There's no one to talk about my pain at home.	1	2	3	4
5. I will pay attention to my family members when they are in trouble.	1	2	3	4
6. I'm self-centered and I don't care about my family.	1	2	3	4
7. We participate in things we are all interested in.	1	2	3	4
8. We don't express our love for each other.	1	2	3	4

9. We can share the housework together.	1	2	3	4
10. We prefer to do things separately rather than with the whole family.	1	2	3	4
11. We will discuss and consult together when we encounter problems.	1	2	3	4
12. My family and I don't care about each other.	1	2	3	4
13. We can support each other in times of crisis.	1	2	3	4
14. It's hard to talk to my family when I come across something that makes me sad.	1	2	3	4
15. I will take the initiative to talk to my family.	1	2	3	4
16. My family members have a cold war with each other .	1	2	3	4
17. My family members can be modest to each other when there is a conflict in the family.	1	2	3	4
18. My family members always get along with each other.	1	2	3	4
19. My family members often quarrel with each other.	1	2	3	4
20. I can tell my family about my difficulties and troubles.	1	2	3	4
21.I don't talk to my family when I'm angry.	1	2	3	4
22. My family members only care about themselves and ignore the family.	1	2	3	4
23. We'll show each other our love.	1	2	3	4
24. I seldom consider other family members' opinions when I do things.	1	2	3	4
25. We will discuss the division of housework.	1	2	3	4
26. We take turns to share different housework in the family.	1	2	3	4
27.We don't have enough time to get along and communicate with each other.	1	2	3	4
28. I am very satisfied that my family members spend time with me.	1	2	3	4
29. I will support the ideas or decisions of other family members.	1	2	3	4
30. I choose to take it alone when I have something to worry about.	1	2	3	4
31. I communicate with my family members as soon as possible when something happens to me.	1	2	3	4
32. I feel like not to stay at home.	1	2	3	4
33. I feel that everyone in my family is backing each other.	1	2	3	4
34. My family members don't have to be careful when they communicate with each other.	1	2	3	4
35. My family members complain about each other when things go wrong.	1	2	3	4
36. I never tell my family what's on my mind.	1	2	3	4
37. I can help my family members when they are in trouble.	1	2	3	4
38. We will listen to each others' opinions when we meet problems.	1	2	3	4
39. There is little time for my family members to spend time with each other.	1	2	3	4
40. We all share family obligations.	1	2	3	4
41. We are willing to spend a lot of energy doing things at home.	1	2	3	4
42. I will try my best to spend time with my family members.	1	2	3	4
43. I will care for my family members.	1	2	3	4
44. I don't tell my family what happened.	1	2	3	4
45. I will discuss the solution with my family if I have a problem.	1	2	3	4
46. My family members are seldom gentle and considerate to each other.	1	2	3	4
47. I don't feel stressed at home.	1	2	3	4
48. My family members love each other.	1	2	3	4
49. My family members often blame and criticize each other.	1	2	3	4
50. I can give warmth and comfort to my family members when they need it.	1	2	3	4

51. My family members take part in recreational activities together.	1	2	3	4
52. Everyone in my family does his/her job.	1	2	3	4
53. We share interesting stories together.	1	2	3	4
54. There is always full of laughter among my family members at home.	1	2	3	4
55. We do housework together.	1	2	3	4
56. We seldom have family conflicts.	1	2	3	4

3 The Contrast of the Number of between the Initial Questionnaire and the Formal Questionnaire.

Q1	Q2	Q3	Q4	Q5	Q6	Q7	Q8	Q12	Q13	Q14	Q15	Q16	Q17
1	2	3	4	5	6	7	8	9	10	11	12	13	14
Q18	Q19	Q20	Q21	Q22	Q23	Q24	Q26	Q27	Q28	Q29	Q31	Q32	Q33
15	16	17	18	19	20	21	22	23	24	25	26	27	28
Q34	Q35	Q36	Q37	Q38	Q39	Q40	Q42	Q43	Q44	Q45	Q46	Q47	Q48
29	30	31	32	33	34	35	36	37	38	39	40	41	42
Q49	Q50	Q51	Q52	Q53	Q54	Q55	Q56	Q57	Q59	Q60	Q61	Q62	Q63
43	44	45	46	47	48	49	50	51	52	53	54	55	56

Note: Q1, Q2，Q3…Q63 represent the serial number of the initial questionnaire; 1, 2, 3…56 represent the serial numbers of the formal questionnaire.
